# Mechanistic Pathways Underlying Breastfeeding Challenges Following Cesarean Delivery: A Systematic Review

**DOI:** 10.3390/nu18172911

**Published:** 2026-09-04

**Authors:** Ray Wagiu Basrowi, Febriansyah Darus, I Gusti Ayu Nyoman Partiwi, Hilna Khairunisa Shalihat, Refani Alycia Kusuma, Dessy Pratiwi

**Affiliations:** 1 Occupational Medicine Division, Department of Community Medicine, Faculty of Medicine, Universitas Indonesia, Jakarta 10320, Indonesia; 2 Danone Specialized Nutrition Indonesia, Jakarta 12940, Indonesia; 3 Health Collaborative Center, Jakarta 12430, Indonesia; 4 Department of Obstetric Gynecology, Gatot Subroto Army Hospital (RSPAD), Jakarta 10410, Indonesia; febriansyah.darus@gmail.com; 5 Bunda Mother and Child Hospital (RSIA Bunda), Jakarta 10350, Indonesia; ayupartiwi@gmail.com; 6 Faculty of Medicine, Universitas Sumatera Utara, Medan 20155, Indonesia; shalihahilna@gmail.com; 7 Adam Malik Hospital, Medan 20136, Indonesia; 8 Department of Nutrition, Faculty of Medicine, Universitas Negeri Semarang, Semarang 50229, Indonesia; refani.alycia15@gmail.com

**Keywords:** cesarean delivery, breastfeeding challenges, breastfeeding self-efficacy, skin-to-skin contact, lactation support

## Abstract

**Background/Objectives**: Cesarean delivery has been linked with breastfeeding difficulties. However, the mechanistic pathways underlying these challenges remain incompletely understood. This systematic review aimed to synthesize evidence regarding the physiological, clinical, psychological, and health system-related mechanisms contributing to breastfeeding challenges following cesarean delivery. **Methods**: A systematic search was conducted in PubMed/MEDLINE and Scopus for studies published between 2020 and 2026. Studies with observational, interventional, and qualitative designs that examined breastfeeding challenges following cesarean delivery were eligible. A theory-informed narrative synthesis was applied, categorizing challenges into physiological, clinical, psychological, and health system domains and integrating them into a pathway-based conceptual framework. **Results**: Twenty-six studies were included. Across heterogeneous study designs, commonly reported breastfeeding challenges included delayed lactogenesis, postoperative pain and functional limitations, difficulties with early mother–infant interaction, concerns regarding human milk sufficiency, reduced breastfeeding self-efficacy, and variability in postpartum breastfeeding support. These findings were organized into physiological, clinical, psychological, and health-system domains. Cross-study synthesis suggested potential interactions among these domains, particularly involving delayed breastfeeding initiation, breastfeeding difficulties, lactation-related concerns, maternal perceptions, and subsequent feeding decisions. However, the proposed relationships were not uniformly tested within individual studies. Intervention studies suggested that strategies including skin-to-skin contact, breastfeeding counseling, and enhanced lactation support may improve selected breastfeeding outcomes. **Conclusions**: The findings suggest that breastfeeding challenges following cesarean delivery may involve interconnected biological, behavioral, clinical, and health-system factors. Understanding these mechanisms is essential for developing supportive and context-sensitive interventions that optimize breastfeeding establishment while maintaining adequate infant nutrition.

## 1. Introduction

Breastfeeding is universally recognized as the optimal source of infant nutrition, providing essential nutrients, immunological protection, and long-term health benefits for both infants and mothers [1]. According to the World Health Organization, breastfeeding should commence within the first hour of birth and be exclusively administered for the first six months of life. Breastfeeding should be continued until the infant reaches two years of age or older [2,3]. Despite these well-established benefits, global breastfeeding practices remain suboptimal. According to the United Nations Children’s Fund (UNICEF) in 2023, only 48% of infants under six months are exclusively breastfed, which is insufficient to meet international objectives and underscores the persistent disparities in maternal and child health interventions [4]. Furthermore, early initiation of breastfeeding remains limited, with only approximately 46% of newborns breastfed within the first hour of life, a critical determinant of successful lactation [4,5].

Parallel to these challenges, the global rate of cesarean delivery has increased substantially, accounting for approximately 21% of births worldwide, with considerable variation across countries and regions [6]. A new study indicates that cesarean section is consistently linked to a decreased likelihood of exclusive breastfeeding and a shortened duration of breastfeeding. Recent epidemiological studies have shown that infants born via cesarean delivery have significantly lower odds (OR ≈ 0.68) of exclusive breastfeeding compared to those born vaginally [7]. This association reflects not only clinical factors but also a complex interplay of physiological, behavioral, and health system-related mechanisms.

Cesarean delivery has been proposed to influence physiological processes involved in lactation initiation, including oxytocin- and prolactin-related pathways [8]. Vaginal birth stimulates oxytocin and prolactin release, facilitating early lactogenesis and mother–infant bonding, whereas cesarean delivery, particularly when performed under general anesthesia, can delay these processes [9]. It contributes to delayed lactogenesis II, reduced milk production, and difficulties in early breastfeeding establishment [10]. In addition, the mother’s inability to move, post-operative pain, and delayed skin-to-skin contact delay the initiation of breastfeeding. Psychological factors, including maternal tension, anxiety, and decreased breastfeeding self-efficacy, are more frequently reported among women who undergo cesarean delivery, which exacerbates these physiological barriers [11,12,13].

Beyond individual-level mechanisms, health system factors also play a critical role. Clinical practices such as mother–infant separation, delayed rooming-in, and insufficient lactation support in postoperative care settings exacerbate breastfeeding challenges [14]. Evidence indicates that cesarean delivery frequently results in delayed breastfeeding initiation and reduced breastfeeding duration, primarily as a result of these systemic and clinical barriers [13,15]. These challenges are not isolated but interact synergistically, creating a cascade of difficulties that can ultimately influence maternal decision-making regarding infant feeding.

Previous studies and reviews have established an association between cesarean delivery and less favorable breastfeeding outcomes, including delayed initiation, reduced exclusivity, and shorter breastfeeding duration [16,17]. However, the evidence describing how these outcomes may emerge remains fragmented. The existing literature has generally examined individual factors such as delayed lactogenesis, postoperative pain, breastfeeding self-efficacy, or healthcare practices separately, rather than considering their potential interactions during the early postpartum period [11,16,18]. Moreover, the extent to which these factors represent findings directly demonstrated by individual studies versus mechanisms inferred across studies has not been clearly distinguished.

Breastfeeding challenges following cesarean delivery should be understood within the broader context of maternal recovery and infant nutritional adequacy. Delayed lactogenesis, postoperative pain, maternal fatigue, and disrupted early breastfeeding interactions may temporarily compromise breastfeeding establishment during the early postpartum period [10,11,12,13]. Breast-milk substitute use has also been reported as a feeding response in some studies; however, the determinants, clinical indications, and appropriateness of supplementation were beyond the scope of this review [15]. This perspective highlights the importance of understanding breastfeeding challenges not merely as isolated difficulties, but as interconnected mechanisms operating within broader biological, behavioral, and systemic pathways.

Therefore, this systematic review synthesizes contemporary evidence published from 2020 to early 2026 to identify recurring physiological, clinical, psychological, and health-system factors associated with breastfeeding challenges after cesarean delivery and to explore how these factors may interact during breastfeeding establishment. Through a cross-study synthesis of heterogeneous evidence, the review distinguishes primary-study findings from review-level interpretations while developing a hypothesis-generating framework that highlights potential pathways and modifiable intervention points. This approach is intended to complement previous association-focused literature by providing a contemporary, mechanism-informed synthesis while avoiding causal claims that cannot be supported by the heterogeneous evidence base. By integrating findings across heterogeneous study designs, the review seeks to develop a hypothesis-generating framework that identifies potentially modifiable intervention points while clearly distinguishing primary-study evidence from review-level interpretation. The study was supported by Danone Specialized Nutrition Indonesia, and two authors are employees of the funding organization. These relationships were transparently considered when interpreting findings related to infant feeding responses, including breast-milk substitute use.

## 2. Materials and Methods

### 2.1. Systematic Literature Review

The conduct and reporting of this review were in accordance with the Preferred Reporting Items for Systematic Reviews and Meta-Analyses (PRISMA) statement. The review protocol was registered in the International Prospective Register of Systematic Reviews (PROSPERO; CRD420261373848). The completed PRISMA 2020 Checklist is provided as Supplementary Table S2.

### 2.2. Search Strategy and Eligibility Criteria

A systematic literature search was conducted to identify studies examining mechanistic pathways underlying breastfeeding challenges following cesarean delivery. The search was performed in electronic databases, PubMed/MEDLINE and Scopus, covering studies published between January 2020 and March 2026. The final search was completed on 13 April 2026. The search strategy combined controlled vocabulary (e.g., MeSH and Emtree terms) and free-text keywords related to cesarean delivery, breastfeeding challenges, lactation difficulties, and breastfeeding support. The full search strategies for each database are provided in Supplementary Table S1.

The 2020 starting point was selected to focus the synthesis on contemporary evidence and recent developments in postpartum breastfeeding care, including early skin-to-skin contact, rooming-in, breastfeeding counseling, lactation support, and cesarean-specific postoperative care. Earlier systematic and narrative reviews were considered to contextualize the evidence gap, but earlier primary studies were not included in the present synthesis. This approach was intended to provide a contemporary evidence map rather than a historical review of breastfeeding after cesarean delivery. However, restricting the search period to 2020 onward may have excluded earlier primary studies that contributed to the understanding of lactation physiology and breastfeeding barriers, and this limitation was considered when interpreting the findings.

Studies were eligible if they involved postpartum women who underwent cesarean delivery and reported breastfeeding challenges or lactation-related difficulties. Observational, interventional, qualitative, and mixed-methods studies published in peer-reviewed journals in English were included. Reviews, editorials, conference abstracts, case reports, and non-human studies were excluded.

### 2.3. Study Selection and Data Extraction

All retrieved records were imported into Mendeley Desktop (version 1.19.8; Mendeley Ltd., London, UK) for reference management, and duplicates were removed. Two reviewers independently screened titles, abstracts, and full-text articles according to predefined eligibility criteria. Discrepancies were resolved through discussion and consensus.

Data were independently extracted by two reviewers using a standardized form. Extracted information included study characteristics, participant characteristics and sample size, type of cesarean delivery where reported, breastfeeding-related challenges and indicators, relevant maternal, infant, clinical, psychological, and health-system factors, evidence type, and study-reported breastfeeding-related findings. Study-reported breastfeeding-related findings were extracted as reported in the individual primary studies and were subsequently distinguished from potential mechanistic relationships developed through the cross-study synthesis.

### 2.4. Quality Assessment

Methodological quality and risk of bias were assessed using design-specific appraisal tools. Randomized controlled trials (RCTs) were evaluated using the Cochrane Risk of Bias 2 (RoB 2) tool, observational studies were assessed using the Newcastle–Ottawa Scale (NOS), mixed-methods studies were appraised using the Mixed Methods Appraisal Tool (MMAT), qualitative studies were assessed using the Critical Appraisal Skills Programme (CASP) checklist, and quasi-experimental studies were evaluated using the Joanna Briggs Institute (JBI) critical appraisal checklist. For studies using mixed-methods designs with a clearly defined primary quantitative cohort component, the quantitative component was classified and appraised according to the primary study design. Two reviewers independently conducted the quality assessments, and any discrepancies were resolved through discussion and consensus. The appraisal results were used to inform the interpretation of individual study findings and the overall evidence base, rather than to infer causal certainty of the pathways identified through cross-study synthesis.

### 2.5. Data Synthesis

Due to heterogeneity in study designs, definitions of breastfeeding challenges, and reported measures, a meta-analysis was not conducted. Instead, a theory-informed narrative synthesis was performed. Findings were categorized into physiological, clinical, psychological, and health system-related domains to identify mechanistic pathways underlying breastfeeding challenges following cesarean delivery.

## 3. Results

### 3.1. Study Selection

**Figure 1:**
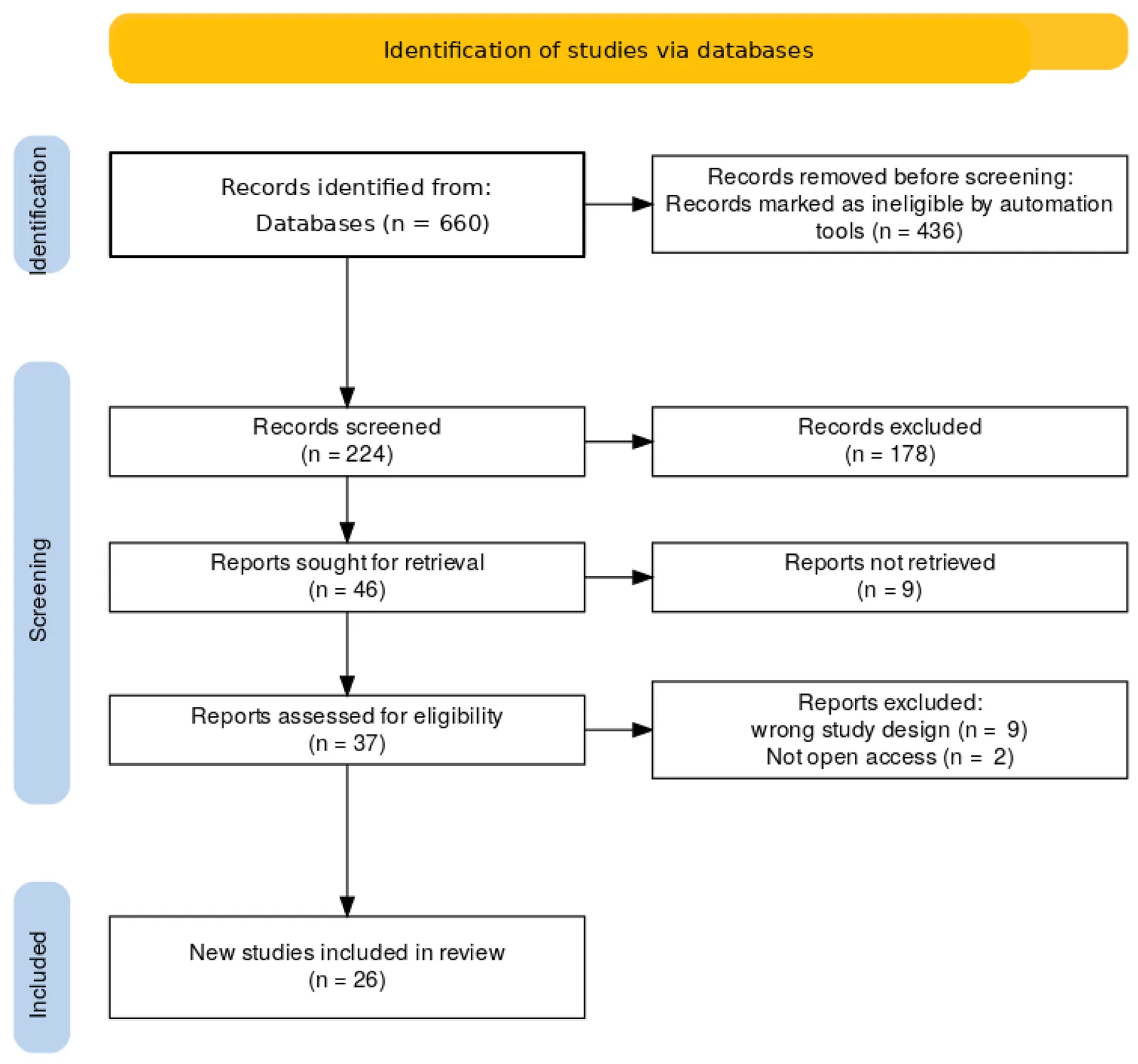
PRISMA Flowchart.

A total of 660 records were identified through searches of PubMed/MEDLINE and Scopus. Following removal of 436 records, 224 records underwent title and abstract screening. Of these, 178 records were excluded, and 46 reports were sought for retrieval. Nine reports could not be retrieved, and 37 full-text reports were therefore assessed for eligibility. Eleven reports were subsequently excluded based on the predefined eligibility criteria, resulting in 26 studies included in the final synthesis (Figure 1). The included studies comprised observational designs (cross-sectional, cohort, and case–control), randomized controlled trials, and qualitative or mixed-methods studies, reflecting the multidimensional nature of breastfeeding challenges following cesarean delivery.

### 3.2. Characteristics of Included Studies

The characteristics of the included studies are summarized in Table 1. The studies were conducted across diverse geographical settings, including countries in Asia, Europe, Africa, Australia, and the Americas. Observational designs constituted the largest proportion of the evidence base (*n* = 18), including cross-sectional studies [19,20,21,22,23,24], prospective or retrospective cohorts [25,26,27,28,29,30,31,32,33,34], and case–control designs [35,36]. Furthermore, four randomized controlled trials (RCTs) evaluated interventions designed to support breastfeeding establishment and reduce breastfeeding challenges following cesarean delivery [37,38,39,40]. Qualitative and mixed-methods studies provided complementary evidence on maternal experiences, contextual barriers, and breastfeeding support, while a quasi-experimental study evaluated a supportive postpartum intervention [41,42,43].

Sample sizes varied substantially, ranging from small qualitative samples (*n* = 19) [42] to large population-based datasets exceeding 3000 participants [21,24]. Postpartum women who underwent cesarean deliveries, including elective and emergency procedures, comprised the primary study populations. However, certain studies also included comparisons with vaginal deliveries. Across studies, breastfeeding-related measures and lactation indicators were heterogeneous, including exclusive breastfeeding (EBF), mixed feeding, use of breast-milk substitutes, breastfeeding initiation, breastfeeding duration, and breastfeeding effectiveness scores (e.g., LATCH, BBAT). This variability contributed to the decision to perform a narrative synthesis.

The different study designs contributed distinct forms of evidence to the synthesis. Observational studies primarily provided evidence on associations and potential predictors of breastfeeding challenges, whereas randomized controlled and quasi-experimental studies provided evidence regarding selected breastfeeding-support interventions. Qualitative and mixed-methods studies contributed contextual and experiential evidence concerning maternal experiences and healthcare-related barriers. Accordingly, these forms of evidence were considered according to their respective methodological characteristics and were not treated as equivalent when developing the proposed cross-study pathways.

### 3.3. Study-Reported Breastfeeding Challenges

The included studies reported a range of breastfeeding-related challenges following cesarean delivery. Because the studies differed in design, outcome definitions, and variables assessed, the four domains presented below should be interpreted as an analytic framework rather than as domains uniformly examined in every study. Table 2 maps study-reported factors to these domains.

#### 3.3.1. Physiological and Lactation-Related Factors

Several studies reported lactation-related difficulties following cesarean delivery, including delayed lactogenesis, reduced human milk production, and difficulties establishing effective milk transfer [19,20,27,33]. Some studies discussed alterations in hormonal or neuroendocrine processes, particularly involving oxytocin and prolactin, in relation to delayed onset of lactation [20,40]. However, these physiological processes were not directly assessed in all studies, and in some cases the proposed mechanisms were inferred from reported breastfeeding or lactation-related findings. Impaired infant feeding responses and delayed mother–infant interaction were also reported as factors associated with breastfeeding difficulties in several studies [25,27]. Insufficient human milk supply was reported in some studies and was associated with earlier breastfeeding cessation [21,32]. Accordingly, these findings support the presence of recurrent physiological and lactation-related challenges following cesarean delivery, while the relative contribution and causal role of individual physiological mechanisms remain uncertain.

#### 3.3.2. Clinical and Functional Factors

Clinical and functional difficulties reported across studies were closely related to postoperative recovery following cesarean delivery. These included postoperative pain, fatigue, limited maternal mobility, positioning difficulties, delayed breastfeeding initiation, and delayed skin-to-skin contact [20,31,34,41]. Several studies reported associations between these factors and difficulties with infant positioning, latch, or early breastfeeding initiation [23,35]. Delayed skin-to-skin contact was also reported in relation to cesarean delivery and was associated with less favorable early breastfeeding establishment in some studies [24,34,43]. The individual contribution of postoperative pain, mobility limitations, breastfeeding initiation, and skin-to-skin contact could not be consistently separated from other maternal, infant, procedural, and healthcare-related factors across studies. Therefore, these findings are interpreted as potentially interacting clinical and functional factors rather than independently established causal mechanisms.

#### 3.3.3. Psychological and Behavioral Factors

Psychological and behavioral factors reported in the included studies included low breastfeeding self-efficacy, anxiety, stress, depressive symptoms, concerns regarding human milk sufficiency, and negative birth experiences [28,33,37,39]. Several studies reported associations between breastfeeding self-efficacy, perceived human milk insufficiency, and breastfeeding continuation or cessation [21,32,38]. Negative birth experiences and emotional distress were also described in relation to breastfeeding motivation and persistence [41,42]. Temporal relationships between psychological factors and breastfeeding difficulties were not consistently established across studies. Therefore, these factors are interpreted as potential interacting or modifying factors within the breastfeeding establishment process rather than as established mediators.

#### 3.3.4. Health-System and Care-Delivery Factors

Health-system and care-delivery factors reported across the included studies included variability in lactation support, inconsistent clinical practices, delayed rooming-in, mother–infant separation, inconsistent implementation of Baby-Friendly Hospital Initiative (BFHI) practices, and gaps in postpartum breastfeeding education and continuity of care [22,26,34,41]. Several studies described variability in postoperative breastfeeding support and mother–infant contact within hospital settings [24,43,44]. Gaps in breastfeeding education and continuity of care were also reported, particularly in resource-limited settings [33,36]. These findings suggest that the organization and delivery of postpartum care may influence breastfeeding establishment following cesarean delivery. However, the extent to which individual health-system factors contribute to breastfeeding difficulties varied across settings and could not be consistently separated from maternal, clinical, and infant-related factors.

### 3.4. Cross-Study Synthesis of Potential Pathways

The cross-study synthesis suggested several potential pathways linking cesarean delivery with breastfeeding challenges during the early postpartum period (Table 3). These pathways were not uniformly tested within individual studies; most studies examined selected components of the proposed relationships. The pathways presented in this section therefore represent review-level interpretations developed by integrating converging findings across heterogeneous study designs rather than established causal mechanisms.

One recurring pattern involved delayed breastfeeding initiation and subsequent breastfeeding difficulties. Several studies reported associations between delayed initiation, breastfeeding problems, or lactation insufficiency and less favorable breastfeeding-related measures [20,21,24]. These findings support individual components of a potential pathway in which disruption of early breastfeeding establishment may be followed by difficulties with effective breastfeeding. The complete sequence, from cesarean delivery through early breastfeeding disruption to subsequent feeding patterns, was not directly tested in most studies.

A second potential pathway involved postoperative and functional factors. Postoperative pain, fatigue, limited mobility, and delayed skin-to-skin contact were reported across several studies and were associated with difficulties in positioning, latch, or breastfeeding initiation [20,24]. These findings suggest that postoperative recovery may represent an important context in which breastfeeding challenges develop. The contribution of individual clinical factors cannot be isolated consistently from maternal, infant, procedural, and healthcare-related factors across the available evidence.

Psychological and health-system factors were also reported in association with breastfeeding difficulties. Low breastfeeding self-efficacy, anxiety, concerns regarding human milk sufficiency, variable lactation support, mother–infant separation, and delayed rooming-in were described across different studies [37,39]. These factors may interact with physiological and clinical difficulties during the early postpartum period. The available evidence does not consistently establish the temporal ordering among these factors, and they are therefore interpreted as potential interacting or modifying factors rather than confirmed mediators.

The role of breast-milk substitutes should also be interpreted within the context of infant nutritional adequacy. When breastfeeding capacity is temporarily compromised during postoperative recovery or delayed lactogenesis, breast-milk substitutes may serve as a context-dependent strategy to help maintain adequate infant nutritional intake [26,37,38,44].

Evidence from intervention studies provides support for the potential modifiability of selected components of this framework. Breastfeeding counseling, early skin-to-skin contact, and enhanced postpartum lactation support were associated with improvements in breastfeeding establishment or reductions in breastfeeding difficulties in selected studies [37,38,44]. The variation in intervention characteristics and outcome assessment means that these findings do not establish a single optimal intervention pathway, but they identify potentially modifiable points within the broader process.

The framework summarizes associations and contextual relationships reported across the included studies and integrates them at the review level. Arrows indicate hypothesized or potential relationships derived from cross-study synthesis and do not represent demonstrated causal pathways or formal mediation effects. Breast-milk substitute use is shown only as a feeding response reported in some studies and is not presented as an intervention or recommendation. The framework should therefore be interpreted as hypothesis-generating rather than as a validated causal model.

**Figure 2:**
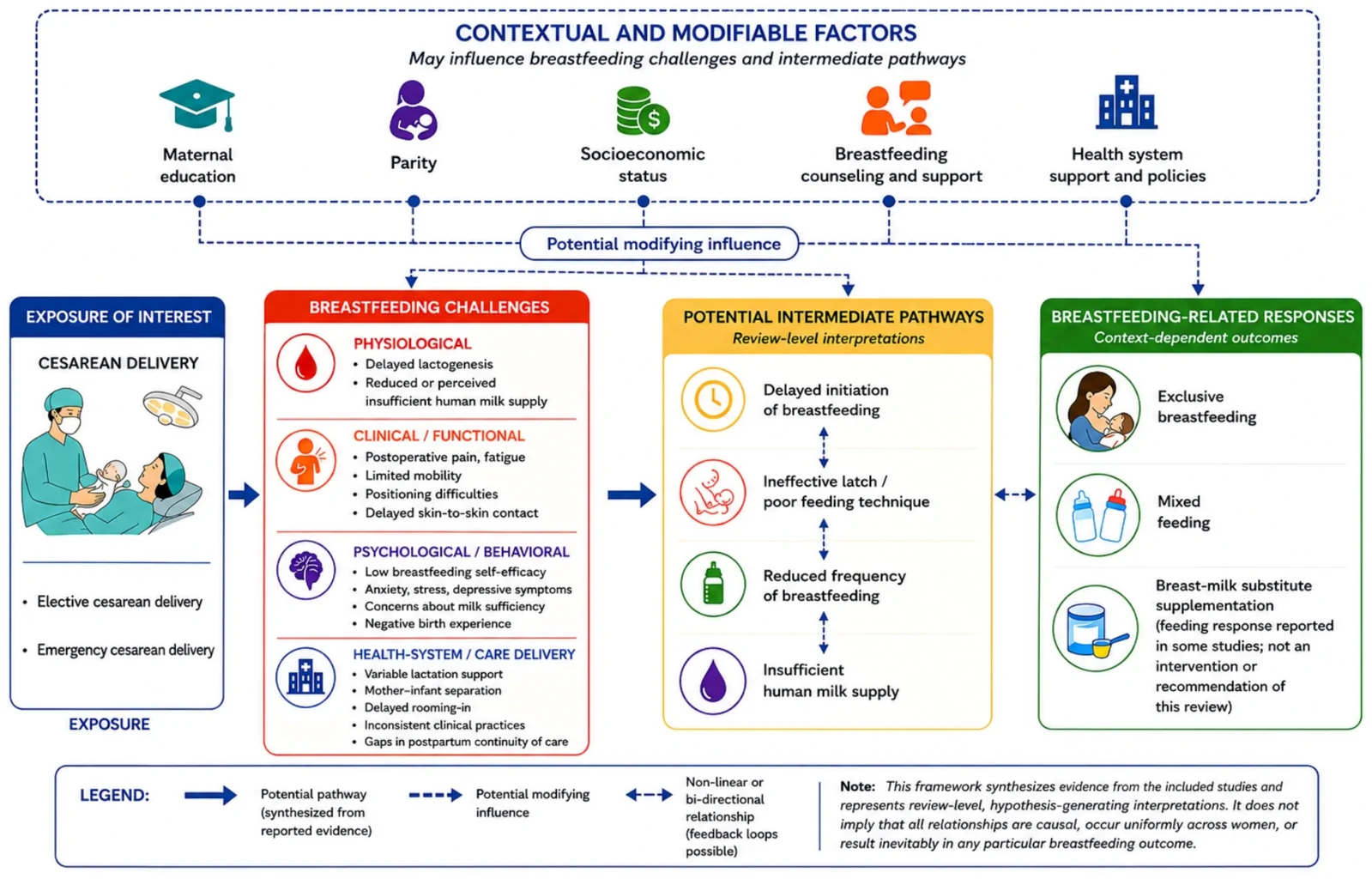
Hypothesis-generating framework of potential relationships linking cesarean delivery with breastfeeding challenges and breastfeeding-related responses.

Based on these cross-study findings, we propose a hypothesis-generating framework in which cesarean delivery and associated perioperative circumstances may be linked with breastfeeding challenges through interacting physiological, clinical, psychological, and health-system pathways (Figure 2). The framework distinguishes reported findings from review-level interpretation and is intended to guide future longitudinal and causal research. It does not imply that the proposed sequence occurs uniformly across women or that cesarean delivery inevitably results in breastfeeding failure.

### 3.5. Quality Assessment

The methodological quality and risk of bias of the included studies were assessed using design-specific appraisal tools (Table 4, Table 5, Table 6, Table 7 and Table 8). Most observational studies assessed using the Newcastle–Ottawa Scale (NOS) were classified as low risk of bias, while a smaller number were rated as moderate risk because of limitations in comparability, selection, or outcome assessment [19,20,21,22,23,24,25,26,27,28,29,30,31,32,33,34,35,36]. The four randomized controlled trials were assessed using the Cochrane Risk of Bias 2 (RoB 2) tool and were generally judged to have low risk of bias, although some concerns were identified in selected domains for one trial. Mixed-methods studies were appraised using the Mixed Methods Appraisal Tool (MMAT [41,43], the qualitative study using the Critical Appraisal Skills Programme (CASP) checklist [42], and the quasi-experimental study using the Joanna Briggs Institute (JBI) critical appraisal checklist [44]. These assessments supported interpretation of individual study findings but did not establish the certainty or causality of the proposed pathways. Given the predominance of observational evidence and heterogeneity across study designs, the proposed mechanistic relationships are interpreted as hypothesis-generating rather than confirmed causal mechanisms.

## 4. Discussion

This review extends the previous literature by moving beyond the description of associations between cesarean delivery and breastfeeding-related indicators and by synthesizing potential mechanisms through which breastfeeding challenges may develop during the early postpartum period. The cross-study synthesis identified recurring physiological, clinical and functional, psychological and behavioral, and health-system factors across heterogeneous study designs. Rather than treating these domains as independently established mediators, the present review proposes that they may interact during breastfeeding establishment. This distinction is important because most included studies examined individual components of these relationships rather than testing an integrated mechanistic pathway. The principal contribution of this review is the integration of study-reported findings into a hypothesis-generating framework while explicitly distinguishing empirical findings from review-level interpretation. Within this framework, cesarean delivery is considered the primary exposure, breastfeeding challenges represent potential intermediate processes, and breastfeeding-related responses represent observations that may arise from multiple interacting maternal, infant, clinical, psychological, and health-system factors [16].

The added value of this review lies in three aspects. First, it integrates physiological, clinical, psychological, and health-system factors that have often been examined separately in previous studies. Second, it distinguishes empirical findings reported by individual studies from mechanistic relationships inferred through cross-study synthesis, an important distinction given the predominance of observational and heterogeneous evidence. Third, it translates these findings into a hypothesis-generating framework that identifies potentially modifiable points during the early postpartum period. This framework does not replace evidence from longitudinal or experimental studies; rather, it provides a structured basis for testing whether the proposed interactions and temporal sequences are supported in future research.

From a biological perspective, delayed lactogenesis II emerged as a recurrent lactation-related factor across the included evidence [45]. Delayed transition from colostrum production to established human milk production has been discussed in relation to differences in neuroendocrine signaling, including oxytocin- and prolactin-related processes, as well as perioperative factors such as surgical stress, blood loss, anesthesia, and delayed mother–infant contact [46,47,48,49]. Importantly, these physiological processes were not directly measured in all included studies. In several studies, hormonal or lactation mechanisms were proposed to explain observed breastfeeding difficulties rather than directly tested. Accordingly, delayed lactogenesis should be interpreted as a potential biological pathway or contributing factor identified through synthesis rather than as an established mediator of the effect of cesarean delivery [10].

Several studies also reported associations between delayed lactogenesis or perceived insufficient human milk supply and subsequent breastfeeding difficulties or supplementation [50,51,52]. These findings suggest a potential sequence in which difficulties in establishing human milk production may influence maternal perceptions and breastfeeding-related responses. The available evidence does not establish that this sequence occurs uniformly, nor does it allow the relative contribution of physiological and perceptual components to be separated consistently.

Importantly, the review did not evaluate the efficacy, safety, medical indications, or appropriateness of breast-milk substitute supplementation. Breast-milk substitute use is therefore interpreted only as a feeding response reported in some studies and should not be considered an intervention or recommendation arising from this review [53]. Its occurrence may reflect a range of clinical, maternal, infant, and contextual circumstances surrounding early breastfeeding establishment. This distinction is particularly important when interpreting feeding responses in studies of women following cesarean delivery.

From a clinical and functional perspective, postoperative pain, fatigue, limited mobility, delayed recovery, and delayed skin-to-skin contact were repeatedly reported in relation to difficulties with positioning, latch, breastfeeding initiation, or breastfeeding frequency [11,54,55,56,57]. The available evidence indicates that postoperative functional factors, including pain, fatigue, mobility limitations, and difficulties with positioning or latch, are repeatedly reported in relation to breastfeeding difficulties, although their contribution cannot be separated consistently from the broader clinical circumstances surrounding cesarean delivery [23,27]. Additionally, early skin-to-skin contact represents one potentially modifiable component of this pathway. Several studies reported lower rates or delayed implementation of skin-to-skin contact following cesarean delivery, while other evidence linked early mother–infant contact with breastfeeding establishment [58,59]. Rather than indicating that cesarean delivery inevitably disrupts these processes, the findings suggest that perioperative care practices may modify the extent to which postoperative difficulties are experienced during early breastfeeding. This distinction shifts the interpretation from delivery mode as a deterministic barrier toward the potentially modifiable conditions surrounding recovery and early breastfeeding support.

Psychological and behavioral factors may provide another pathway through which physical and clinical difficulties are translated into breastfeeding-related responses. Across the included studies, low breastfeeding self-efficacy, anxiety, and perceived or insufficient human milk supply were repeatedly reported among women experiencing breastfeeding difficulties following cesarean delivery. Emerging evidence using structural equation modeling has examined relationships among maternal attitudes, perceptions, intentions, and breastfeeding-related responses. Although these analyses provide information about relationships among psychological and behavioral variables, they do not necessarily establish causal ordering [60,61]. These observations are broadly compatible with behavioral frameworks such as the Theory of Planned Behavior, in which intention, perceived control, and confidence may influence health-related behavior [62,63]. Selected intervention studies included in this review reported improvements in breastfeeding-related measures following counseling or psychosocial support, suggesting that these approaches may represent potentially modifiable components of postpartum breastfeeding care [62]. The potential role of psychological factors may therefore lie not only in their association with breastfeeding difficulties but also in their interaction with physiological and clinical conditions during postpartum recovery.

The direction of these relationships also remains uncertain. For example, low breastfeeding self-efficacy may contribute to breastfeeding difficulties, but experiencing breastfeeding difficulties may also reduce maternal confidence. Similarly, perceived insufficient milk supply may precede changes in feeding practices but may also be influenced by breastfeeding frequency and other postpartum experiences. Longitudinal studies are therefore needed to clarify the temporal relationships among these factors.

From a health-system perspective, the synthesis indicates that breastfeeding establishment occurs within a care environment that may either facilitate or constrain early mother–infant interaction. Mother–infant separation, delayed rooming-in, variable lactation support, and inconsistent implementation of early breastfeeding support practices were reported across several studies [64,65,66]. Evidence concerning early breastfeeding, rooming-in, and skin-to-skin contact further suggests that postpartum care practices may be associated with lactation establishment. These findings suggest that breastfeeding challenges following cesarean delivery may reflect not only maternal and infant factors but also characteristics of the postpartum care environment [67,68,69]. Recent digital trend analyses further suggest that breastfeeding support accessibility and informational needs remain important concerns among mothers, emphasizing the growing relevance of accessible and responsive breastfeeding support systems in contemporary settings [70]. Evidence concerning Baby-Friendly Hospital Initiative practices also supports the relevance of early mother–infant contact, rooming-in, and breastfeeding support to breastfeeding initiation and exclusivity [71,72]. These findings suggest that health-system factors may interact with physiological, clinical, and psychological factors rather than operate as isolated determinants. The proposed framework therefore places healthcare delivery alongside maternal and biological factors as part of a broader system surrounding breastfeeding establishment following cesarean delivery.

An important consideration is confounding by indication. Women undergoing cesarean delivery, particularly emergency or medically indicated procedures, may differ from those delivering vaginally in maternal and infant characteristics that also influence breastfeeding, including obesity, gestational diabetes, preterm birth, neonatal morbidity, and mother–infant separation. Therefore, observed associations cannot necessarily be attributed to delivery mode alone, and residual confounding may remain despite statistical adjustment in some studies [30,73]. In addition, most included studies examined individual components of the proposed relationships rather than the complete sequence within a longitudinal model, making temporal ordering and reverse causation difficult to establish. The proposed relationships should therefore be interpreted as potentially interconnected processes and hypotheses for further testing rather than as an established causal pathway.

The cross-study synthesis suggests that breastfeeding challenges following cesarean delivery may develop through a sequence of partially overlapping processes rather than through a single, uniform pathway. Early postoperative and clinical factors, including pain, fatigue, limited mobility, and delayed skin-to-skin contact, may coincide with delayed breastfeeding initiation or difficulties with positioning and latch. These difficulties may occur alongside delayed lactogenesis, reduced or perceived insufficient human milk supply, and concerns regarding milk sufficiency, while psychological and health-system factors may influence how these challenges are experienced and managed [13,33,74,75]. The available evidence generally examined individual components of these relationships rather than testing the complete sequence within a single analytical model. The framework proposed in this review therefore represents a review-level synthesis of plausible relationships rather than a validated mediation model or demonstrated causal pathway.

The synthesis also identifies potentially modifiable points within the proposed pathway. Evidence from selected intervention studies suggests that early skin-to-skin contact, breastfeeding counseling, lactation support, and appropriate postpartum care may help reduce breastfeeding challenges and support breastfeeding establishment following cesarean delivery [38,39,44]. These findings indicate potential opportunities for intervention at multiple levels, although differences in intervention characteristics, populations, and assessment methods prevent identification of a single optimal intervention strategy. The value of the proposed framework therefore lies in identifying plausible relationships and potential intervention points for future testing rather than establishing a fixed causal sequence.

Several strengths and limitations should be considered when interpreting the findings. The inclusion of diverse study designs allowed the review to capture different forms of evidence, including associations from observational studies, intervention evidence from randomized or quasi-experimental studies, and contextual or experiential evidence from qualitative research. This methodological diversity was valuable for developing a broad cross-study interpretation but also introduced substantial heterogeneity in study populations, measurement of breastfeeding challenges, definitions of breastfeeding-related indicators, and analytical approaches. This heterogeneity limited the feasibility and appropriateness of quantitative meta-analysis. Individual odds ratios, adjusted odds ratios, hazard ratios, and qualitative findings were therefore not pooled or ranked against one another. A large study-specific effect estimate should not be interpreted as evidence of a stronger or more important mechanistic pathway, because effect magnitude is influenced by study design, outcome definition, adjustment strategy, sample size, and model specification. In addition, the predominance of observational evidence means that residual confounding and reverse causation cannot be excluded, while the qualitative and mixed-methods evidence provides contextual depth but cannot establish temporal or causal relationships. Because the review did not estimate a common pooled effect, a formal quantitative certainty-of-evidence assessment such as GRADE was not undertaken. The proposed mechanistic pathways should therefore be interpreted as hypothesis-generating rather than confirmatory.

Within these limitations, the review contributes a contemporary synthesis of evidence published from 2020 to early 2026 and provides a structured framework for understanding how physiological, clinical, psychological, and health-system factors may interact during breastfeeding establishment following cesarean delivery. The framework identifies potentially modifiable intervention points while explicitly acknowledging uncertainty in the temporal and causal relationships among these factors. Future longitudinal, mediation, and intervention studies should test the proposed relationships directly and determine whether the temporal and contextual interactions identified in this review are reproducible across different healthcare settings.

## 5. Conclusions

This review synthesizes contemporary evidence suggesting that breastfeeding challenges following cesarean delivery cannot be adequately understood through delivery mode alone. Across heterogeneous study designs, physiological, clinical, psychological, and health-system factors were reported in association with breastfeeding establishment and continuation.

Based on the cross-study synthesis, we propose a hypothesis-generating framework in which postoperative functional difficulties, early breastfeeding disruption, lactation-related concerns, maternal psychological responses, and healthcare practices may interact during the early postpartum period. These relationships were not uniformly tested within individual studies and should therefore not be interpreted as established causal or mediating mechanisms. The proposed framework should be considered a synthesis-level interpretation and requires testing in longitudinal and causal studies with appropriate consideration of clinical context and potential confounding.

The principal contribution of this review is the integration of fragmented contemporary evidence into a mechanism-informed framework that distinguishes study-reported findings from review-level interpretations and identifies potentially modifiable points during early postpartum care. Given the predominance of observational evidence and heterogeneity in study populations, exposures, and outcome measures, the framework should guide rather than replace empirical testing. Future longitudinal studies using formal mediation and causal modelling are needed to establish temporal relationships, account for relevant maternal and infant clinical factors, and determine which intervention points may most effectively support breastfeeding after cesarean delivery.

## Figures and Tables

**Table 1:** Characteristics of Included Studies.

No	Author (Year)	Title	Country	Study Design	Sample Size	Population	Type of Cesarean
1	Porożyńska & Szablewska (2026) [19]	Factors Related to Pregnancy and Childbirth and Their Relationship with Exclusive Breastfeeding—A Cross Sectional Study	Poland	Cross-sectional	557	Mothers with infants aged 6–12 months	CS vs. Vaginal
2	Chen et al. (2026) [20]	Determinants of postpartum lactation insufficiency in caesarean section in Chinese mothers: A cross-sectional study	China	Cross-sectional	265	CS mothers	Mixed CS
3	Özer Aslan et al. (2025) [25]	Impact of Delivery Method on Initiation and Continuation of Breastfeeding: A Prospective Cohort Study	Turkey	Prospective cohort	338	Mothers and infants	Planned & Emergency CS
4	Farasati et al. (2024) [37]	The Effect of Home Counseling on Breastfeeding Self-efficacy and Breastfeeding Performance Following Cesarean Section	Iran	RCT	60 (Intervention *n* = 30; Control *n* = 30)	Primiparous CS mothers	Mixed (elective and emergency CS, balanced allocation)
5	Latorre et al. (2024) [26]	The effect of on-site and on-call nurse on exclusive breastfeeding in two different hospital settings: a prospective observational cohort study	Italy	Prospective cohort	564	Mother–infant dyads	Mixed CS
6	Perrella et al. (2024) [41]	Australian Women’s Experiences of Establishing Breastfeeding after Caesarean Birth	Australia	Mixed-methods	961	Post-CS women	Elective CS & Non-elective CS
7	Chen et al. (2023) [21]	Factors associated with exclusive breastfeeding during postpartum in Lanzhou city, China: a cross-sectional study	China	Cross-sectional	3738	Postpartum women	Mixed CS (Cesarean vs. vaginal delivery comparison; elective/emergency not specified)
8	Singh et al. (2023) [27]	Association of Caesarean delivery and breastfeeding difficulties during the delivery hospitalization: a community-based cohort of women and full-term infants in Alberta, Canada	Canada	Prospective cohort	418	Mothers and infant’s dyads	Planned/unplanned CS
9	Titaley et al. (2023) [24]	The multiple factors of suboptimal early feeding practices among infants aged 0–5 months in Indonesia	Indonesia	Cross-sectional	3198	Infants aged 0–5 months	CS vs. Vaginal
10	Igarashi et al. (2023) [44]	Effectiveness of an Early Skin-to-Skin Contact Program for Pregnant Women with Cesarean Section: A Quasi-Experimental Trial	Tanzania	Quasi-experimental	172 (86 intervention, 86 control)	CS mothers	Mixed CS (planned and emergency)
11	Takács et al. (2022) [28]	Emergency cesarean section is a risk factor for depressive symptoms when breastfeeding is limited	Czech Republic	Prospective cohort	404 (6 weeks), 234 (9 months postpartum)	Postpartum women	Planned CS vs. Emergency CS
12	Jarrett et al. (2022) [35]	Factors contributing to neonatal readmissions to a level 4 hospital within 28 days after birth	Australia	Case–control, retrospective cross-sectional study	129 readmitted neonates and 122 controls	Neonates & mothers	Mixed (emergency and elective CS included but not primary exposure)
13	Martin et al. (2022) [29]	Cesarean section and breastfeeding outcomes in an Indigenous Qom community with high breastfeeding support	Argentina	Prospective longitudinal cohort with mixed- methods	89	Indigenous mothers-infants	Mixed (scheduled and unscheduled CS)
14	Zimmerman et al. (2022) [22]	Breastfeeding challenges and support in a high initiation population	Israel	Cross-sectional	868	Postpartum mothers with infants ≤ 3 months	CS vs. Vaginal
15	Sokou et al. (2022) [30]	Breastfeeding in Neonates Admitted to an NICU: 18-Month Follow-Up	Greece	Cohort	279	Mothers and neonates (NICU)	CS vs. Vaginal
16	Mena-Tudela et al. (2022) [31]	Is Early Initiation of Maternal Lactation a Significant Determinant for Continuing Exclusive Breastfeeding up to 6 Months?	Spain	Retrospective Cohort	342	Mothers and newborns	CS vs. Vaginal
17	Karaahmet & Bilgiç (2022) [38]	Breastfeeding success in the first 6 months of online breastfeeding counseling after cesarean delivery and its effect on anthropometric measurements of the baby: a randomized controlled study	Turkey	RCT	151	Primiparous CS mothers	Elective CS
18	Rahman et al. (2022) [36]	Long-term effects of caesarean delivery on health and behavioural outcomes of the mother and child in Bangladesh	Bangladesh	Case–control	600	Mothers and children’s dyads	Mixed CS
19	Saddki et al. (2022) [32]	Determinants of non-exclusive breastfeeding practice during the first 6 months after an elective caesarean birth: a prospective cohort study	Malaysia	Prospective cohort	171	Elective CS mothers	Elective CS
20	Lian et al. (2022) [33]	Determinants of delayed onset of lactogenesis II among women who delivered via Cesarean section at a tertiary hospital in China: a prospective cohort study	China	Prospective cohort	468	CS women	Mixed CS (elective and emergency)
21	Johar et al. (2021) [34]	Factors Associated with Early Breastfeeding Initiation among Women Who Underwent Cesarean Delivery at Tertiary Hospitals in Kelantan, Malaysia	Malaysia	Prospective cohort	171	Mothers with elective CS	Elective CS
22	Wen et al. (2021) [39]	Effects of a theory of planned behavior-based intervention on breastfeeding behaviors after cesarean section: A randomized controlled trial	China	RCT	132 (66 intervention, 66 control)	Elective CS mothers	Elective CS
23	Cirpanli & Hicyilmaz (2020) [23]	Postcesarean Difficulties and their Association with Breastfeeding Success in Postpartum Women	Turkey	Cross-sectional	220	CS mothers	CS only
24	Wen et al. (2020) [42]	An exploration of the breastfeeding behaviors of women after cesarean section: A qualitative study	China	Qualitative	19	CS mothers	Mixed CS (planned + emergency)
25	Wang et al. (2020) [40]	Impact of Intraoperative Infusion and Postoperative PCIA of Dexmedetomidine on Early Breastfeeding After Elective Cesarean Section: A Randomized Double-Blind Controlled Trial	China	RCT	160	Elective CS mothers	Elective CS
26	Hernández-Cordero et al. (2020) [43]	Barriers and facilitators to breastfeeding during the immediate and one-month postpartum periods, among Mexican women: a mixed methods approach	Mexico	Mixed-methods	543	Postpartum mothers	CS vs. Vaginal

**Table 2:** Study-Reported Breastfeeding Challenges and Related Factors Classified by Review-Defined Domains.

No	Author (Year)	Physiological	Clinical	Psychological	Health System	Study-Reported Breastfeeding-Related Findings
1	Porożyńska & Szablewska (2026) [19]	Delayed lactogenesis, postpartum complications	Early lactation problems (latch, milk supply), mode of delivery	Birth satisfaction (not significant)	Early practices (skin-to-skin, timing) were not significant	Early lactation problems were associated with lower EBF.
2	Chen et al. (2026) [20]	Delayed lactogenesis, low milk volume, hormonal disruption	Postpartum pain, gestational diabetes, high BMI	Postpartum depression	Lack of early breastfeeding support (skin-to-skin)	Lactation insufficiency prevalence 48.3% with multiple independent risk factors.
3	Özer Aslan et al. (2025) [25]	Impaired infant reflexes, lack of oxytocin stimulation, delayed lactation	Planned & emergency CS, anesthesia, post-operative pain	Maternal fatigue, early bonding is disturbed	SSC practice is not optimal without BFHI intervention	Cesarean delivery was associated with delayed breastfeeding initiation; the study reported that the difference diminished over time.
4	Farasati et al. (2024) [37]	Insufficient milk supply	Infant refusal to latch, feeding difficulty	Low breastfeeding self- efficacy (BSE), stress	Lack of post-discharge support (before intervention)	The intervention group had fewer breastfeeding problems than the comparison group.
5	Latorre et al. (2024) [26]	Perceived low milk supply	Nipple fissures, latch difficulties	Low maternal confidence, knowledge gap	Inadequate breastfeeding support (on-call vs. on-site nurse)	Lack of continuous support leads to early breastfeeding difficulties and discontinuation.
6	Perrella et al. (2024) [41]	Pain, reduced mobility, fatigue	Difficulty positioning, inability to pick up baby, delayed initiation	Stress, anxiety, negative birth experience	Limited staff support, rushed care, conflicting advice, restricted partner access	Breastfeeding challenges were reported in relation to care-related and birth-experience factors.
7	Chen et al. (2023) [21]	Delayed milk secretion due to hormonal/stress response	Post-cesarean pain, slow recovery, physical limitation	Anxiety, negative attitude, Insufficient milk supply	Limited targeted postpartum support, education gaps	Insufficient milk supply was reported by 57% of participants; the study examined its relationship with EBF cessation.
8	Singh et al. (2023) [27]	Delayed lactogenesis II, low milk supply	Post-surgical recovery, pain, delayed skin-to-skin	Low breastfeeding self- efficacy, perception of insufficient milk	Limited early support (rooming-in, lactation support variability)	Cesarean delivery was associated with low milk supply (aOR 1.62) and infant feeding-related difficulties (aOR 1.33).
9	Titaley et al. (2023) [24]	Disrupted lactogenesis (post-CS hormonal pathway)	CS delivery, delayed initiation, prelacteal feeding	Not explicitly dominant	Health facility variation, marketing formula, lack of BF support	Suboptimal feeding was reported in 78.6% of participants, with factors described across multiple levels.
10	Igarashi et al. (2023) [44]	Delayed initiation, mother–infant separation, lack of SSC	CS (planned & emergency), postoperative recovery	Low bonding & delivery satisfaction	Hospital system separates mother and baby after CS, lack of SSC implementation	The study reported higher delivery satisfaction and shorter hospitalization among participants receiving SSC.
11	Takács et al. (2022) [28]	Not explicitly assessed/reported	Emergency CS-related complications	Increased stress, vulnerability to depression	Not explicitly assessed/reported	Emergency CS is associated with lower likelihood of exclusive breastfeeding.
12	Jarrett et al. (2022) [35]	Low milk supply, dehydration-related issues	Poor latch, feeding inefficiency, weight loss	Maternal inexperience (primiparous	Early discharge, limited follow-up support	Feeding difficulties are the leading cause (72.9%) of neonatal readmission.
13	Martin et al. (2022) [29]	Delayed lactogenesis, low milk onset	Pain, mobility limitation, latch problems	Anxiety, lack of experience (primiparous mothers)	Strong kin/community support, postpartum assistance	Early breastfeeding problems occur but are resolved with strong social support.
14	Zimmerman et al. (2022) [22]	Insufficient milk supply (18.5%)	Mechanical problems (latch, nipple pain) (55.2%)	Maternal concern, low confidence, first-time motherhood	Inadequate access to breastfeeding support (hospital & community)	BF difficulties were commonly reported and were associated with earlier breastfeeding cessation.
15	Sokou et al. (2022) [30]	Prematurity, immature suck– swallow coordination	NICU admission, tube feeding, delayed initiation	Maternal stress, anxiety, reduced confidence	Mother–infant separation, NICU environment, limited access	NICU admission and prematurity were associated with shorter breastfeeding duration and lower exclusivity.
16	Mena-Tudela et al. (2022) [31]	Post-surgical recovery, delayed lactogenesis post-surgical recovery, delayed lactogenesis	Cesarean section, analgesia, delayed SSC, low LATCH score	Fatigue, anxiety, negative birth experience	Delayed skin-to-skin contact (SSC), hospital practice	Low LATCH score (<9) was associated with earlier cessation of exclusive breastfeeding in the study.
17	Karaahmet & Bilgiç (2022) [38]	Post-cesarean pain, nipple problems	Incorrect positioning, poor latch	Low self-efficacy, perception of insufficient milk	Lack of support without counseling	The counseling intervention was associated with reduced breastfeeding challenges and improved breastfeeding initiation.
18	Rahman et al. (2022) [36]	Post-surgical pain, headache, hip pain	Physical limitations, reduced functional ability	Not explicitly assessed	Limited postpartum care access (LMIC context)	Cesarean delivery was associated with breastfeeding problems (OR 3.19).
19	Saddki et al. (2022) [32]	Insufficient milk supply	Breast pain, difficulty positioning	Low BF self-efficacy	Lack of structured lactation support & education	Perceived milk insufficiency was frequently reported among participants who did not exclusively breastfeed.
20	Lian et al. (2022) [33]	Delayed lactogenesis, low albumin (nutritional status), hormonal dysregulation	Postoperative pain, delayed initiation, low breastfeeding frequency	Postpartum depression (EPDS ≥ 10)	Use of breast-milk substitutes, low early breastfeeding stimulation	DOLLI was associated with nutritional factors, breastfeeding frequency, and postpartum depression.
21	Johar et al. (2021) [34]	Pain, fatigue, nausea, breast pain, perceived low milk	Post-surgical recovery, limited mobility, infant sleepiness, poor latch	Low confidence, worry, perception of insufficient milk	Skin-to-skin contact (SSC), BFHI practices, staff support	Early initiation was associated with SSC, infant alertness, and feeding technique.
22	Wen et al. (2021) [39]	Postoperative pain, delayed lactogenesis, infant sucking disorders	CS section, medication use, delayed initiation	Low self-confidence, low breastfeeding intention	Lack of ongoing education & family support	The TPB-based intervention was associated with higher EBF and fewer reported lactation problems.
23	Cirpanli & Hicyilmaz (2020) [23]	Insufficient milk supply, no milk production	Latching difficulty, positioning problems, nipple issues, engorgement	Lack of experience, low confidence	Limited nurse support, delayed SSC, workload constraints	Breastfeeding problems were reported as important barriers to breastfeeding success, particularly difficulties with latching, nipple problems, and positioning.
24	Wen et al. (2020) [42]	Incision pain, back pain, lactation delay	Poor positioning, nipple damage, limited mobility	Low confidence, depressive mood, ambivalence	Conflicting advice, lack of structured education, cultural pressure	Breastfeeding behavior was described in relation to biological, psychological, and sociocultural factors.
25	Wang et al. (2020) [40]	Reduced prolactin & oxytocin due to stress	Postoperative pain, delayed lactation	Anxiety, depression, reduced breastfeeding motivation	Limited perioperative breastfeeding optimization	Postoperative pain and negative mood were associated with delayed breastfeeding initiation and lactation difficulties.
26	Hernández-Cordero et al. (2020) [43]	Insufficient milk supply, sore nipples	CS recovery, delayed initiation, use of breast-milk substitutes	Belief milk is insufficient, low confidence, cultural beliefs	Belief milk is insufficient, low confidence, cultural beliefs	The study reported hospital practices and breast-milk substitute marketing as contextual factors related to breastfeeding.

Note: The physiological/lactation-related, clinical/functional, psychological/behavioral, and health-system/care-related domains were defined a priori by the review authors for synthesis purposes. Entries within each domain represent factors explicitly reported or analyzed in the corresponding primary studies; factors not reported or assessed were not inferred from the study context. The domain classification does not imply that the primary study conceptualized these factors within the four-domain framework. The “Study-reported breastfeeding-related findings” column summarizes findings reported by the respective primary studies and does not represent causal interpretations generated by the review authors.

**Table 3:** Study-Reported Findings and Hypothesis-Generating Pathways Identified Through Cross-Study Synthesis.

No.	Author (Year)	Study-Reported Findings	Evidence Type	Potential Pathway Identified Through Cross-Study Synthesis	Interpretive Role
1	Porożyńska & Szablewska (2026) [19]	Delayed lactation and breastfeeding difficulties were reported following cesarean delivery; early lactation success was associated with breastfeeding status.	Observational	Cesarean delivery → early lactation difficulties → breastfeeding establishment	Supports a potential association between early lactation difficulties and subsequent breastfeeding establishment; the complete pathway was not directly tested.
2	Chen et al. (2026) [20]	Delayed lactogenesis, pain, and endocrine-related factors were reported in association with breastfeeding difficulties.	Observational	Perioperative factors → delayed lactogenesis → breastfeeding difficulties	Suggests a potential physiological pathway; endocrine mechanisms should be interpreted cautiously when not directly measured.
3	Özer Aslan et al. (2025) [25]	Delayed breastfeeding initiation and impaired neonatal feeding responses were reported following cesarean delivery.	Observational	Delayed initiation/infant feeding difficulties → breastfeeding establishment difficulties	Provides evidence for selected components of the proposed pathway rather than the complete causal sequence.
4	Farasati et al. (2024) [37]	The evaluated intervention was associated with changes in breastfeeding self-efficacy and breastfeeding difficulties.	RCT	Support intervention → improved self-efficacy → breastfeeding establishment	Provides intervention evidence suggesting that psychological factors may be modifiable.
5	Latorre et al. (2024) [26]	Breastfeeding difficulties and early breastfeeding indicators were reported among women following cesarean delivery.	Observational	Cesarean delivery → breastfeeding difficulties → breastfeeding-related responses	Supports an association but does not establish mediation.
6	Perrella et al. (2024) [41]	Pain and delayed breastfeeding initiation were associated with breastfeeding difficulties; use of breast-milk substitutes was reported in some participants.	Mixed-methods	Postoperative difficulties → delayed breastfeeding establishment → feeding response	Provides evidence for selected components of a potential clinical pathway; the temporal relationship among postoperative difficulties and subsequent feeding responses was not fully established.
7	Chen et al. (2023) [21]	Pain, delayed recovery, and anxiety were associated with breastfeeding difficulties.	Observational	Postoperative recovery + psychological factors → breastfeeding difficulties	Suggests interacting clinical and psychological factors rather than an independently established mediation pathway.
8	Singh et al. (2023) [27]	Delayed lactogenesis, pain, and reduced milk supply were reported following cesarean delivery.	Observational	Perioperative factors → delayed lactogenesis/insufficient milk supply → breastfeeding difficulties	Supports a potential physiological pathway; temporal ordering was not consistently established.
9	Titaley et al. (2023) [24]	Delayed breastfeeding initiation and hormonal-related factors were associated with prelacteal feeding or reduced breastfeeding establishment.	Observational	Delayed initiation → breastfeeding establishment difficulties	Provides evidence for individual components of a potential pathway; the complete sequence was not directly tested.
10	Igarashi et al. (2023) [44]	Mother–infant separation, delayed initiation, and limited skin-to-skin contact were associated with breastfeeding-related measures.	Quasi-experimental	Mother–infant separation/delayed contact → breastfeeding establishment difficulties	Suggests a potential health-system and care-delivery pathway.
11	Takács et al. (2022) [28]	Emergency cesarean delivery was associated with breastfeeding-related psychological symptoms.	Observational	Cesarean-related experience → psychological factors → breastfeeding-related responses	Supports a potential psychological pathway; mediation was not established.
12	Jarrett et al. (2022) [35]	Mode of delivery and breastfeeding-related difficulties were examined in relation to maternal experiences.	Observational	Delivery-related experience → breastfeeding difficulties	Provides contextual evidence for the proposed pathway rather than direct causal evidence.
13	Martin et al. (2022) [29]	Early postpartum breastfeeding problems, including pain and delayed lactation, were reported; social support was also described.	Observational	Postpartum difficulties + social support → breastfeeding establishment	Suggests that clinical and contextual factors may interact during breastfeeding establishment.
14	Zimmerman et al. (2022) [22]	Insufficient human milk supply and maternal concerns were reported in relation to breastfeeding continuation.	Observational	Perceived/insufficient milk supply → breastfeeding-related responses	Supports a potential lactation-related pathway; physiological and perceptual components may not be separable.
15	Sokou et al. (2022) [30]	Prematurity, NICU admission, mother–infant separation, and delayed breastfeeding initiation were associated with breastfeeding difficulties.	Observational	Prematurity/NICU-related factors → separation or delayed initiation → breastfeeding difficulties	Highlights potential confounding by clinical context; the findings should not be interpreted as evidence of an independent effect of cesarean delivery.
16	Mena-Tudela et al. (2022) [31]	Delayed exclusive breastfeeding initiation, reduced skin-to-skin contact, and latch difficulties were reported.	Observational	Delayed initiation/reduced skin-to-skin contact → latch difficulties → breastfeeding establishment	Supports selected components of the proposed clinical pathway.
17	Karaahmet & Bilgiç (2022) [38]	Online breastfeeding counseling improved breastfeeding self-efficacy and breastfeeding-related measures among women following cesarean delivery.	RCT	Breastfeeding counseling → improved self-efficacy → breastfeeding establishment	Provides intervention evidence supporting the potential modifiability of psychological and support-related factors.
18	Rahman et al. (2022) [36]	Pain, physical limitations, and breastfeeding problems were associated with breastfeeding-related measures.	Observational	Postoperative functional difficulties → breastfeeding difficulties	Suggests a clinical/functional pathway; individual effects were difficult to separate.
19	Saddki et al. (2022) [32]	Pain, delayed initiation, insufficient milk supply, and perceived insufficiency were associated with non-exclusive breastfeeding.	Observational	Pain/delayed initiation/milk insufficiency → breastfeeding-related response	Supports individual pathway components but not a complete causal chain.
20	Lian et al. (2022) [33]	Delayed onset of lactogenesis, low breastfeeding frequency, and maternal stress were reported in association with breastfeeding difficulties.	Observational	Delayed lactogenesis + maternal stress → breastfeeding establishment difficulties	Suggests interaction between physiological and psychological factors.
21	Johar et al. (2021) [34]	Poor latch, delayed initiation, and limited breastfeeding support were associated with breastfeeding-related measures.	Observational	Clinical difficulties + support limitations → breastfeeding establishment	Supports the potential interaction of clinical and health-system factors.
22	Wen et al. (2021) [39]	Low self-control/perceived control and breastfeeding support were associated with breastfeeding-related measures.	RCT	Psychological factors + support → breastfeeding establishment	Suggests that behavioral and support-related factors may be modifiable.
23	Cirpanli & Hicyilmaz (2020) [23]	Breastfeeding problems, including latch and milk-supply difficulties, were reported following cesarean delivery.	Observational	Breastfeeding problems → breastfeeding establishment difficulties	Provides evidence for lactation-related components of the proposed pathway.
24	Wen et al. (2020) [42]	Low confidence, poor skills, and breastfeeding problems were associated with breastfeeding-related responses.	Qualitative	Self-efficacy + breastfeeding skills → breastfeeding establishment	Provides contextual evidence for psychological and functional interactions.
25	Wang et al. (2020) [40]	Pain, stress, and delayed lactation were reported in relation to breastfeeding initiation.	RCT	Postoperative recovery + psychological factors → breastfeeding establishment	Suggests potentially modifiable clinical and psychological components, although the individual contributions of these factors were not established.
26	Hernández-Cordero et al. (2020) [43]	Delayed initiation, separation, hospital practices, and formula exposure were reported in relation to breastfeeding establishment.	Mixed-methods	Hospital practices/delayed initiation → breastfeeding establishment	Supports a potential health-system pathway; individual effects were not independently established.

Note: “Study-reported findings” summarizes findings explicitly reported or analyzed in the individual included studies. “Evidence type” indicates the design of the contributing study. The “Potential pathway” column represents a hypothesis-generating interpretation developed through cross-study synthesis and does not indicate that the complete pathway was tested within the individual study. The table is intended to illustrate converging or complementary evidence rather than to rank effect estimates or certainty of evidence. Quantitative effect estimates were not directly compared because of substantial heterogeneity in study designs, populations, exposures, outcomes, analytical approaches, and measurement methods. No formal certainty-of-evidence grading was applied because the synthesis did not estimate a common effect across sufficiently comparable studies. The proposed pathways should therefore not be interpreted as established causal relationships.

**Table 4:** Quality Assessment of Observational Studies Using the Newcastle–Ottawa Scale (NOS).

No	Author (Year)	Study Design	Selection (0–4)	Comparability (0–2)	Outcomes (0–3)	Total Score (0–9)	Risk Category
1	Porożyńska & Szablewska (2026) [19]	Cross-sectional	4	2	3	9	Low Risk of Bias
2	Chen et al. (2026) [20]	Cross-sectional	3	2	3	8	Low Risk of Bias
3	Özer Aslan et al. (2025) [25]	Prospective cohort	4	1	3	8	Low Risk of Bias
4	Latorre et al. (2024) [26]	Prospective cohort	4	2	3	9	Low Risk of Bias
5	Chen et al. (2023) [21]	Cross-sectional	3	2	2	7	Moderate Risk of Bias
6	Singh et al. (2023) [27]	Prospective cohort	4	2	3	9	Low Risk of Bias
7	Titaley et al. (2023) [24]	Cross-sectional	4	2	2	8	Low Risk of Bias
8	Takács et al. (2022) [28]	Prospective cohort	4	2	3	9	Low Risk of Bias
9	Jarrett et al. (2022) [35]	Case–control, retrospective cross-sectional study	4	1	3	8	Low Risk of Bias
10	Martin et al. (2022) [29]	Prospective longitudinal cohort with mixed-methods	4	2	3	9	Low Risk of Bias
11	Zimmerman et al. (2022) [22]	Cross-sectional	3	2	3	8	Low Risk of Bias
12	Sokou et al. (2022) [30]	Cohort	4	2	3	9	Low Risk of Bias
13	Mena-Tudela et al. (2022) [31]	Retrospective cohort	4	2	3	9	Low Risk of Bias
14	Rahman et al. (2022) [36]	Case–control	3	2	2	7	Moderate Risk of Bias
15	Saddki et al. (2022) [32]	Prospective cohort	4	2	3	9	Low Risk of Bias
16	Lian et al. (2022) [33]	Prospective cohort	3	2	3	8	Low Risk of Bias
17	Johar et al. (2021) [34]	Prospective cohort	3	2	3	8	Low Risk of Bias
18	Cirpanli & Hicyilmaz (2020) [23]	Cross-sectional	3	2	3	8	Low Risk of Bias

Note: Methodological quality of observational studies was assessed using the Newcastle–Ottawa Scale (NOS), which evaluates three domains: selection (0–4 points), comparability (0–2 points), and outcome (0–3 points), with a maximum score of 9. Studies were categorized as low (7–9), moderate (5–6), or high risk of bias (<5).

**Table 5:** Risk of Bias Assessment of Randomized Controlled Trials Using the ROB 2 Tool.

No	Author (Year)	Study Design	Randomization	Deviations from Intervention	Missing Outcome Data	Selective Reporting	Overall Risk
1	Farasati et al. (2024) [37]	RCT	Low	Low	Low	Low	Low risk
2	Karaahmet & Bilgiç (2022) [38]	RCT	Low	Low	Low	Low	Low risk
3	Wen et al. (2021) [39]	RCT	Low	Low	Low	Low	Low risk
4	Wang et al. (2020) [40]	RCT	Low	Low	Some concerns	Low	Low risk

Note: Risk of bias for randomized controlled trials was assessed using the Cochrane Risk of Bias 2 (ROB 2) tool across five domains: randomization process, deviations from intended interventions, missing outcome data, measurement of outcomes, and selective reporting. Each domain was rated as “low risk,” “some concerns,” or “high risk,” leading to an overall risk of bias judgment.

**Table 6:** Quality Appraisal of Qualitative Study Using the CASP Checklist.

No	Author (Year)	Study Design	Clear Aim	Appropriate Methodology	Research Design	Recruitment Strategy	Data Collection	Reflexivity	Ethical Issues	Data Analysis	Findings	Value of Research	Overall Judgment
1	Wen et al. (2020) [42]	Qualitative	Yes	Yes	Yes	Yes	Yes	Partial	Yes	Yes	Strong	High	High Quality (Moderate–High confidence)

Note: Methodological quality of qualitative and mixed-methods studies was assessed using the Critical Appraisal Skills Programme (CASP) checklist. Studies were evaluated across domains including research aims, methodology, design, recruitment, data collection, reflexivity, ethical considerations, data analysis, and validity of findings. Overall judgments reflect the rigor, credibility, and relevance of each study.

**Table 7:** Methodological Quality Assessment of Mixed-Methods Studies Using the Mixed Methods Appraisal Tool (MMAT).

No.	Author (Year)	Study Design	Clear Research Question	Data Adequate to Answer Question	Appropriate Mixed-Methods Design	Integration of Qualitative and Quantitative Components	Interpretation of Integrated Results	Divergences/ Inconsistencies Addressed	Quality Criteria Met for Respective Components	Overall Appraisal
1	Perrella et al. (2024) [41]	Mixed-methods	Yes	Yes	Yes	Yes	Yes	Yes	Yes	High quality
2	Hernández-Cordero et al. (2020) [43]	Mixed-methods	Yes	Yes	Yes	Yes	Yes	Yes	Yes	High quality

Note: Methodological quality of mixed-methods studies was assessed using the Mixed Methods Appraisal Tool (MMAT), version 2018. The assessment included two screening questions and five mixed-methods-specific criteria addressing the appropriateness of the mixed-methods design, integration of quantitative and qualitative components, interpretation of integrated findings, consideration of divergences between components, and methodological quality of the individual components.

**Table 8:** Critical Appraisal of the Quasi-Experimental Study Using the JBI Checklist.

No.	Author (Year)	Study Design	Q1	Q2	Q3	Q4	Q5	Q6	Q7	Q8	Q9	Overall Appraisal
1	Igarashi et al. (2023) [44]	Quasi-experimental	Yes	Yes	Unclear	Yes	Yes	Yes	Yes	Yes	Yes	Moderate quality

Note: Q1–Q9 represent the nine domains of the Joanna Briggs Institute (JBI) Critical Appraisal Checklist for Quasi-Experimental Studies. Responses were classified as Yes, No, Unclear, or Not Applicable according to the JBI criteria.

## Data Availability

All data sources used for this study are available online through the PubMed/MEDLINE and Scopus databases.

## Supplementary Materials

The following supporting information can be downloaded at: https://www.mdpi.com/article/10.3390/nu18172911/s1, Table S1: Search Strategy Used for Literature Identification; Table S2: PRISMA 2020 Checklist [76].

## Abbreviations

The following abbreviations are used in this manuscript:

aOR	Adjusted Odds Ratio
BBAT	Bristol Breastfeeding Assessment Tool
BF	Breastfeeding
BFHI	Baby-Friendly Hospital Initiative
BSE	Breastfeeding Self-Efficacy
CASP	Critical Appraisal Skills Programme
CI	Confidence Interval
CS	Cesarean Section
DOLII	Delayed Onset of Lactogenesis II
EBF	Exclusive Breastfeeding
EPDS	Edinburgh Postnatal Depression Scale
HR	Hazard Ratio
LATCH	Latch, Audible swallowing, Type of nipple, Comfort, Hold
NICU	Neonatal Intensive Care Unit
NOS	Newcastle–Ottawa Scale
OR	Odds Ratio
PRISMA	Preferred Reporting Items for Systematic Reviews and Meta-Analyses
PROSPERO	International Prospective Register of Systematic Reviews
ROB 2	Risk of Bias 2 Tool
RR	Relative Risk
SSC	Skin-to-Skin Contact
TPB	Theory of Planned Behavior
