# Supplementary material for: Mechanistic Pathways Underlying Breastfeeding Challenges Following Cesarean Delivery: A Systematic Review
Source: Nutrients. 2026 Sep 4;18(17):2911. doi: 10.3390/nu18172911 (PMC13567548; doi:10.3390/nu18172911)
Supplement: Supplementary file 1 [file nutrients-18-02911-s001.zip › nutrients-4445439-Table S1.pdf]

Supplementary Table S1. Search Strategy Used for Literature Identification

The search strategy combined controlled vocabulary terms (e.g., MeSH terms) and free-text keywords related to cesarean delivery, breastfeeding challenges, lactation difficulties, breastfeeding establishment, breastmilk substitute supplementation, and breastfeeding support. Boolean operators ("AND", "OR") were used to optimize search sensitivity and specificity.

Database	Search Strategy
PubMed	(("Cesarean Section"[Mesh] OR "Cesarean Delivery" OR "Caesarean Section"<br>OR "C-Section" OR cesarean OR caesarean))<br>AND<br>(("Breast Feeding"[Mesh] OR breastfeeding OR "breast feeding" OR lactation OR lactogenesis OR "milk production" OR "breastfeeding establishment"))<br>AND<br>((challenge* OR difficult* OR barrier* OR insufficien* OR "low milk supply" OR "delayed lactogenesis" OR pain OR "skin-to-skin" OR "maternal confidence" OR anxiety OR stress))<br>AND<br>((mechanism* OR pathway* OR mediator* OR process* OR determinant*<br>OR factor*))
Scopus	TITLE-ABS-KEY (("cesarean delivery" OR "caesarean section" OR "c-section" OR cesarean OR caesarean))<br>AND TITLE-ABS-KEY ((breastfeeding OR "breast feeding" OR lactation<br>OR lactogenesis OR "milk production" OR "breastfeeding establishment"))<br>AND TITLE-ABS-KEY ((challenge* OR difficult* OR barrier* OR insufficien* OR "low milk supply" OR "delayed lactogenesis" OR pain<br>OR "skin-to-skin" OR "maternal confidence" OR anxiety OR stress))<br>AND TITLE-ABS-KEY ((mechanism* OR pathway* OR mediator* OR<br>process* OR determinant* OR factor*))

Additional Search Limits

- Publication years: 2020–2026
- Language restriction: English only
- Population: Postpartum women undergoing cesarean delivery
- Study types: Randomized controlled trials, cohort studies, observational studies, qualitative studies, and mixed-methods studies

Additional Identification Methods

Reference list screening of eligible studies was conducted to identify additional relevant articles not captured through electronic database searches.
